# In-Depth Retinal Sensitivity Assessment With the MP3 Type S Microperimeter: A Methods Study

**DOI:** 10.1167/tvst.13.4.14

**Published:** 2024-04-09

**Authors:** Thales A. C. de Guimaraes, Isabela M. C. de Guimaraes, Naser Ali, Angelos Kalitzeos, Michel Michaelides

**Affiliations:** 1 UCL Institute of Ophthalmology, University College London, London, UK; 2 Moorfields Eye Hospital NHS Foundation Trust, London, UK; 3 Universidade Sao Leopoldo Mandic, Campinas, Sao Paulo, Brazil

**Keywords:** MP3, validation, visual field modeling analyzer, microperimetry, fundus-guided perimetry, perimetry

## Abstract

**Purpose:**

Retinal sensitivity is frequently listed as an end point in clinical trials, often with long working practices. The purpose of this methods study was to provide a new workflow and reduced test time for in-depth characterization of retinal sensitivity.

**Methods:**

A workflow for the MP3-S microperimeter with detailed functional characterization of the retina under photopic, mesopic, and scotopic conditions was evaluated. Grids of 32 and 28 test positions for photopic/mesopic and scotopic, respectively, were tested in 12 healthy individuals and compared with an established 68-point grid for test time, mean sensitivity (MS), and bivariate contour ellipse area (BCEA).

**Results:**

The mean test time (range; ±SD) was 10.5 minutes (8.4–14.9; ±2.0) in the 68-point grid and 4.3 minutes (3.8–5.0; ±0.4) in the 32-point grid, which was significantly different (*P* < 0.0001). The mean of difference in test time (±SD; 95% confidence interval) was 6.1 minutes (±2.0; 4.6–7.6). MS and BCEA were significantly correlated between grids (*r* = 0.89 and 0.74; *P* = 0.0005 and 0.014, respectively). Mean test time of subjects who underwent the full protocol (*n* = 4) was 2.15 hours.

**Conclusions:**

The protocol suggested herein appears highly feasible with in-depth characterization of retinal function under different testing conditions and in a short test time.

**Translational Relevance:**

The protocol described herein allows for characterization of the retina under different testing conditions and in a short test time, which is relevant due to its potential for patient prognostication and follow-up in clinical settings and also given its increasing role as a clinical trial end point.

## Introduction

Retinal sensitivity is an important functional parameter that has been increasingly adopted as primary or secondary outcome in clinical trials.^1^^,^^2^ From that perspective, the eye, particularly the retina, is a privileged organ, since retinal function can be measured readily using noninvasive methods.^3^

Historically, Goldmann kinetic perimetry and automated static perimeters, such as the Humphrey Visual Field Analyser (Carl Zeiss, Oberkochen, Germany) and the Octopus 900 (Haag-Streit AG, Köniz, Switzerland), have been used to provide an accurate assessment of the visual field and can detect changes with great standardization. Perimeters such as the Octopus 900 allow for customized grids to be used and the extraction of raw visual field data, which can be readily used for analysis.

Microperimetry—or, more accurately, fundus-guided perimetry—is a psychophysical assessment that probes retinal sensitivity across the macula, arguably providing a more in-depth characterization of the retinal function, particularly of the central section of the retina. This is achieved due to its ability to display and track a live fundus image while adjusting for fixational eye movements, providing an abundance of information on retinal sensitivity, bivariate contour ellipse area (BCEA), and preferred retinal locus (PRL).^1^ Microperimetry devices also allow for functional evaluation of the retina under different lighting conditions. In that regard, the MP3-S is unique as, to the best of our knowledge, it is the only device that allows for testing under three different conditions: (1) photopic, (2) mesopic, and (3) scotopic. Herein we evaluate a new concept for testing with the MP3-S using a less dense grid and more straightforward test methodology, with the purpose of performing a concise and comprehensive functional characterization of the retina.

## Methods

This study adhered to the tenets of the Declaration of Helsinki. Informed consent was obtained for all participants. A full description of our working practice is available in Supplementary Material 1.

### Grid Concept

**Figure 1.**
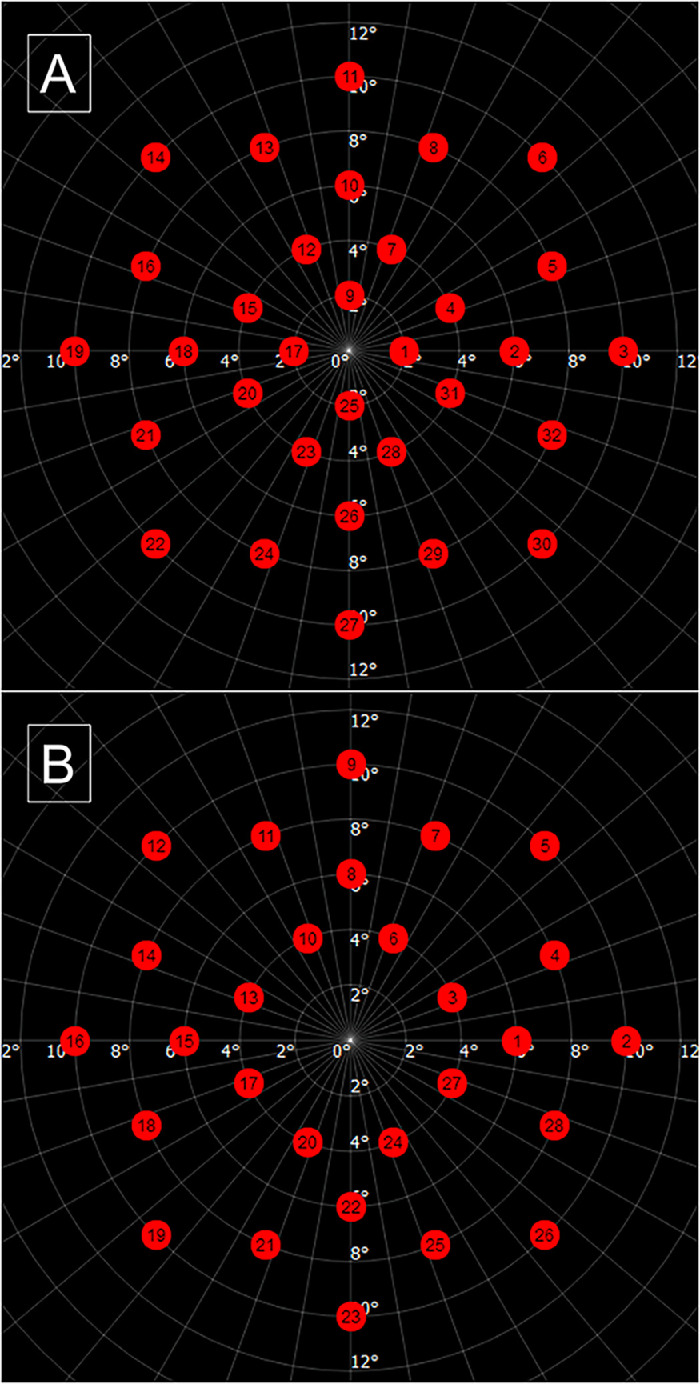
Test grids used for photopic/mesopic (**A**) and scotopic (**B**).

For the purposes of this protocol, we developed two custom grids: a 32-point grid to be used for photopic and mesopic, as well as another 28-point grid to be used in scotopic testing (Fig. 1). Both grids test the central 10 degrees of the macula (10-2). A Goldmann size III stimulus with a 200-ms duration was used in a 4-2 strategy, on a background luminance of 31.4 asb (9.99 cd/m²) for photopic and 4 asb (1.27 cd/m²) for mesopic. The fixation target was a 1-degree size red cross. The dynamic range of the device in these modalities is 34 dB.

In scotopic testing, the grid was composed of 28 points—the same 32 points used in mesopic/photopic testing minus the four positions within 2 degrees of the fovea (rod-free zone; marked as points 1, 9, 17, and 25 in Fig. 1A). A Goldmann size V stimulus with a 200-ms duration in a 4-2 strategy and a background luminance of 0.003 asb (0.0009 cd/m²) was used. Given the presence of the rod-free zone, the fixation target was a white circle with a 3-degree radius. If the subject was unable to see the target, the examiner could change the target to four crosses, and if still not visible, the patient could be guided verbally based on the fundus image on the screen. The dynamic range of the device in the scotopic setting is 24 dB. Both grids are available in the University College London Data Repository (https://doi.org/10.5522/04/25145096.v1).^4^

### Microperimetry Proposed Workflow

For the purpose of this study, we used the MP3-S microperimeter (Nidek, Gamagori, Japan). As aforementioned, this is the only fundus-guided perimetry device that allows accurate determination of retinal sensitivity under three different conditions—namely, (1) photopic, in which the cones contribute to most of the response; (2) mesopic, whereas both population of cells (cones and rods) are active and the response to the stimuli is mixed; and (3) scotopic, in which rod response is isolated after a period of dark adaptation.^1^ Being able to isolate the response of cones and rods is likely to provide further insights into the pathophysiology of retinal disease, particularly when used in combination with retinal imaging techniques.

**Figure 2.**
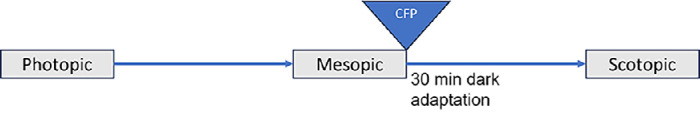
Workflow for the three-modality testing suggested. CFP, color fundus photography.

The workflow we suggest (Fig. 2) consisted of undilated testing in a completely darkened room. Dark adaptation was required only for the scotopic configuration, in which the patient was kept in the room with a blindfold for 30 minutes prior to the test. The protocol consisted of testing the right eye first, followed by the left eye starting with photopic, then mesopic, and, finally, scotopic.

### Grid Validation

With the purpose of comparing the robustness of the 32-point grid against the 68-point grid—since the latter is a commonly used microperimetry grid derived from the Humphrey Visual Field Analyser^5^—we performed both tests in 10 healthy individuals under mesopic conditions. A similar methodology was applied in the ProgSTAR study (S.M.A.R.T. protocol)—a large prospective natural history study in patients with Stargardt disease type I (OMIM #248200)—where the grids used for scotopic had 40 points as opposed to 68 for mesopic testing, the rationale being to reduce patient fatigue during the test, which was in keeping with our purpose.^6^ Only mesopic was used since it is the most commonly used setting, which is universally available across all microperimeters. For the purposes of this validation process, the order of the grids and the eye tested were randomized.

The data were analyzed using two different methods: (1) by comparing the mean sensitivity (MS) in the four central retinal degrees—which we called central macular zone (CMZ)—and between 6 and 10 retinal degrees—peripheral macular zone (PMZ)—and (2) by correlating the overall MS and the BCEA of both grids. Catch trials (false positive [FP] and false negative [FN]) and test time were compared between the two grids. The reliability factor—a metric consisting of the sum of FP and FN divided by the sum of all catch study presentations—was defined with a threshold of <20%. Any test exceeding this would be repeated. No test–retest assessment was undertaken since it was beyond the scope of this study.

### Full Protocol Viability Testing

We have further tested the full methodology consisting of photopic, mesopic and scotopic testing (Fig. 2) in four eyes.

### Statistical Methods

Statistical analysis was performed with the aid of GraphPad Prism V.9 (GraphPad Software, San Diego, CA, USA). Parametric and nonparametric tests were employed, as well as correlation parameters (either Pearson or Spearman, respectively). Significance of all statistical tests was set at *P* < 0.05, and the D'Agostino-Pearson test (omnibus K2) was used to determine normality for all variables. Descriptive statistics were used where relevant.

## Results

### Grid Validation

Ten healthy participants were randomized and tested under mesopic conditions, with a mean age of 31.6 years (range, 24–43), three of whom were male. The contralateral eye was patched throughout the test.

**Figure 3.**
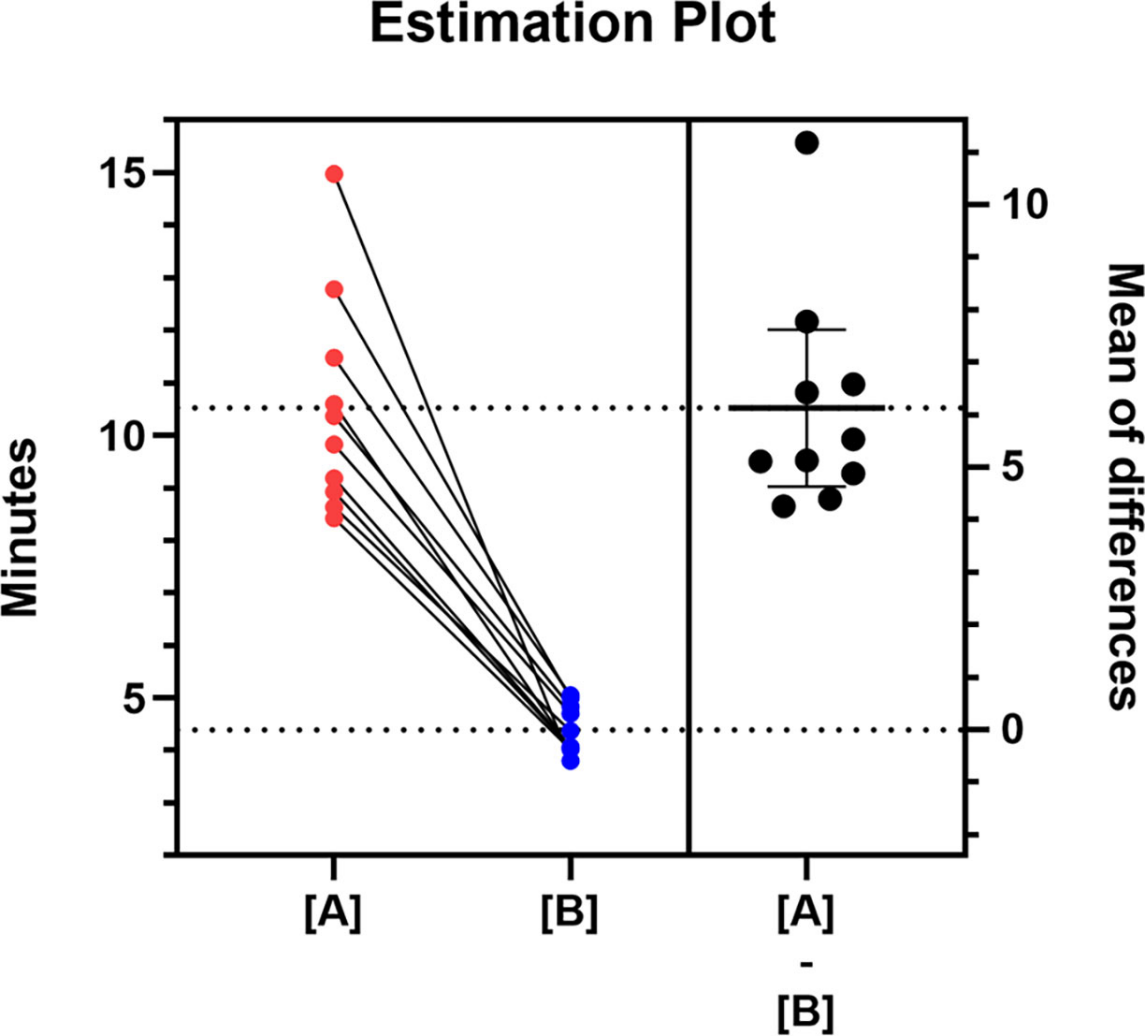
Test time for each subject in the 68-point grid (**A**; *red circles*) and 32-point grid (**B**; *blue circles*), which are connected with a continuous *black line*. On the right, a scatterplot of each patient's test differences, with the corresponding average difference (6.1 minutes) and the 95% CI of the mean time differences between the two tests.

The mean test time (range; ±SD) was 10.5 minutes (8.4–14.9; ±2.0) in the 68-point grid and 4.3 minutes (3.8–5.0; ±0.4) in the 32-point grid, which was significantly different (*P* < 0.0001; *t* = 9.13; *df* = 9; paired *t*-test). The mean of differences in test time (±SD; 95% confidence interval [CI]) between the grids was 6.1 minutes (±2.0; 4.6–7.6). Figure 3 illustrates the estimation plot and mean of differences.

The MS (range; ±SD) was 24.7 dB (21.6–26.7; ±1.4) in the 68-point grid and 24.8 dB (22.6–26.6; ±1.1) in the 32-point grid, which was not significantly different (*P* = 0.40; *t* = 0.89; *df* = 9; paired *t*-test). There was a significant correlation between the MS of both grids (*r* = 0.89; *P* = 0.0005; Pearson correlation coefficient).

**Figure 4.**
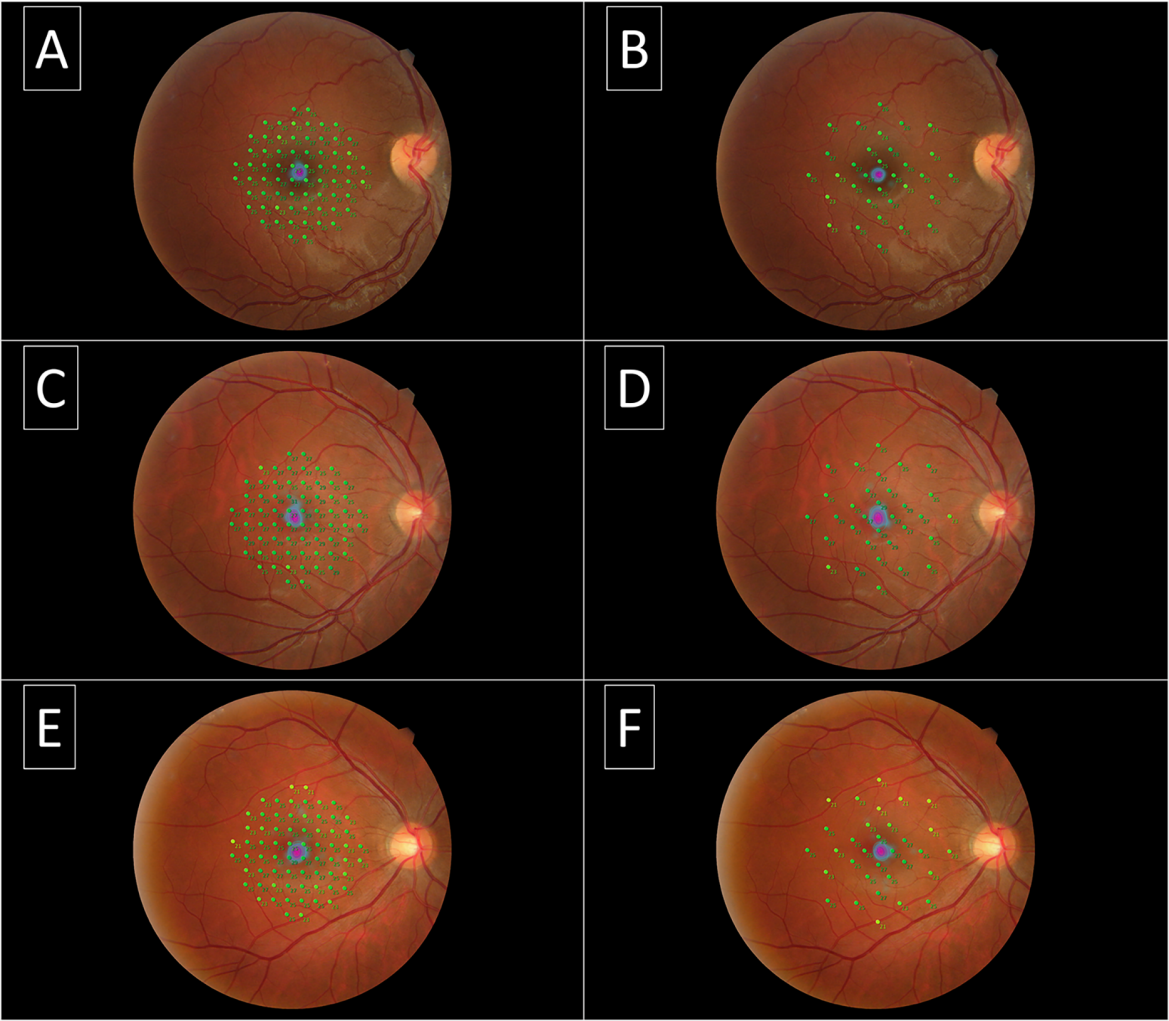
Microperimetry printouts of three subjects. Each column represents the 68-point grid (**A**, **C**, **E**) with the corresponding 32-point grid printouts (**B**, **D**, **F**). The grid is overlaid with a high-resolution pseudo-colour fundus image obtained automatically with the MP3-S at the end of the mesopic testing. The BCEA is represented in the fovea as a heatmap.

**Figure 5.**
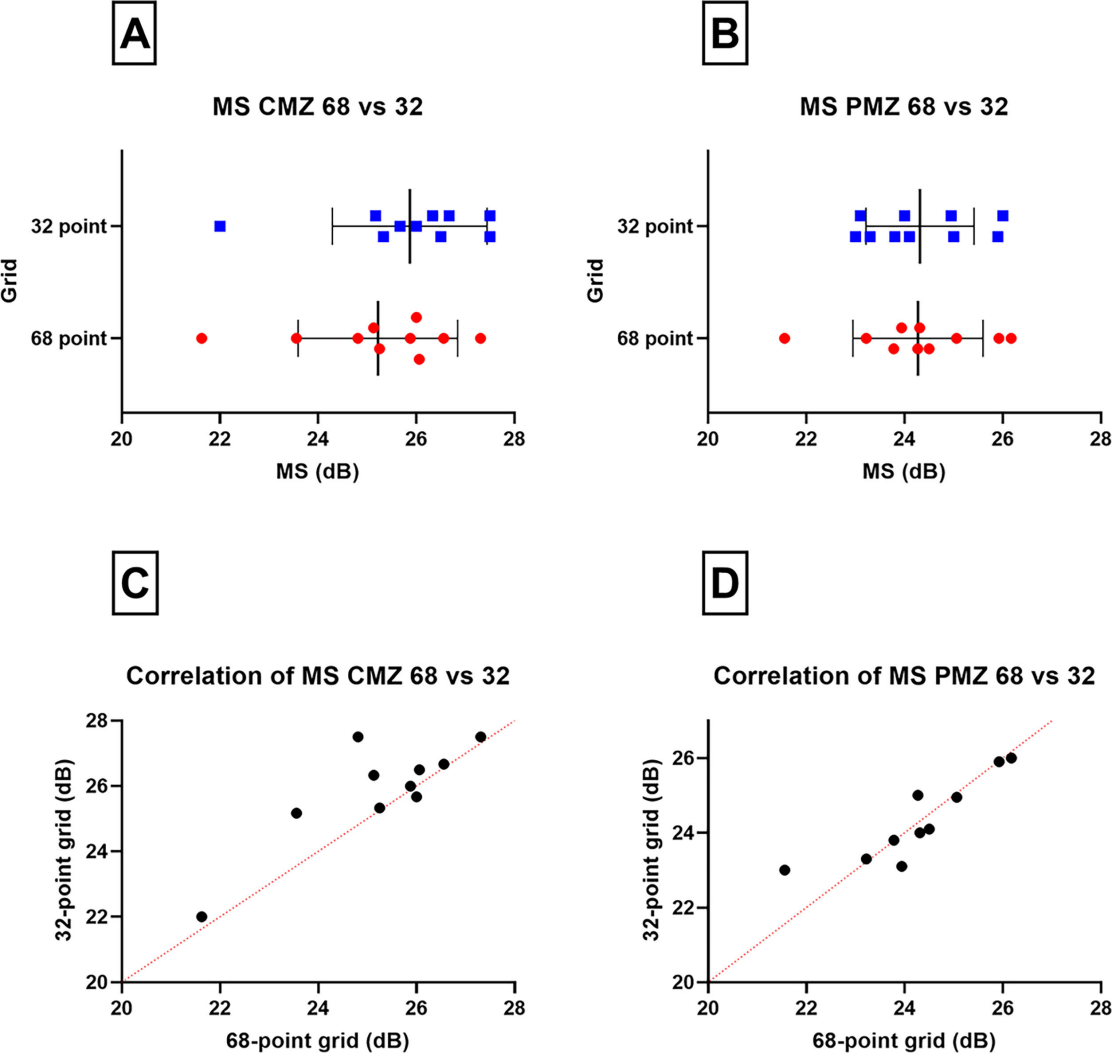
(**A**, **B**) Plot of the MS in the PMZ and CMZ in the 32-point grid (*blue squares*) and the 68-point grid (*red circles*). The *black line* represents the average MS and the whiskers the SD. (**C**, **D**) Correlation of MS in the CMZ and PMZ in each grid, with the *red dashed line* representing the gradient line.

Figure 4 illustrates both tests in three subjects. The average MS (range; ±SD) in the CMZ of the 68- and 32-point grids (Fig. 5A), respectively, was 25.2 dB (21.6–27.3; ±1.6) and 25.8 dB (22–27.5; ±1.5), which was not significantly different (*P* = 0.052; *t* = 2.23; *df* = 9; paired *t*-test). The average MS (range; ±SD) in the PMZ of the 68- and 32-point grids (Fig. 5B), respectively, was 24.2 dB (21.5–26.1; ±1.3) and 24.3 dB (23–26; ±1.1), which was not significantly different (*P* = 0.84; *t* = 0.21; *df* = 9; paired *t*-test). There was a significant intergrid correlation (*r*; *P*; Pearson correlation coefficient) in both the CMZ (Fig. 5C; 0.83; 0.002) and PMZ (Fig. 5D; 0.88; 0.0008).

Similarly, BCEA was also significantly correlated between grids. The mean (range; ±SD) BCEA was 2.52°^2^ (0.8–5.9; ±1.66) in the 68-point grid and 2.15°^2^ (0.5–4.8; ±1.70) in the 32-point grid, which was not statistically different (*P* = 0.36; *t* = 0.96; *df* = 9; paired *t*-test) and significantly correlated (*r* = 0.74; *P* = 0.014; Pearson correlation coefficient).

### Photopic, Mesopic, and Scotopic Retinal Sensitivity

Four healthy eyes were tested with the full workflow (Fig. 2) for photopic, mesopic, and scotopic microperimetry with a mean age (range; ±SD) of 31.0 years (25–36; ±5.7).

The mean photopic, mesopic, and scotopic MS in dB (range; ±SD) was 31.5 (30.9–32.1; ±1.7), 27.8 (27.8–27.8; ±0.2), and 16.7 dB (15.7–17.8; ±1.4). The mean test time of the full protocol was 2.15 hours (range, 1.8–2.25).

## Discussion

In this study, we assessed the feasibility of a novel methodology using the MP3-S microperimeter. This is a medical device with a high tracking speed of 30 Hz coupled with the possibility of testing in all lighting conditions.^1^ This is an improvement over the Macular Integrity Assessment (S-MAIA; CenterVue, Padova, Italy), which, although has a wider dynamic range of 36 dB in both mesopic and scotopic modes, does not perform testing under a photopic background luminance. Similarly, the S-MAIA uses a different concept for scotopic testing, presenting stimuli of two different wavelengths—cyan (505 nm) and red (627 nm)—which was validated in a previous study.^7^ In the MP3-S, scotopic testing is achieved by employing a background luminance of 0.00095 cd/m^2^ without the use of extra filters to attenuate the stimuli.^1^

One intrinsic limitation of traditionally used test grids is that these are overly extensive and burdensome. Given the long testing times and the possibility of being influenced by patient fatigue, we created a custom grid with less test points (in a 4-2 fast strategy) and attempted to correlate with the larger traditional 68-point grid.

What our data suggest is that although the larger grid provides more information due to the intrinsic larger number of test points (more than twice as many)—which is likely necessary for clinical trials when comparing pointwise sensitivity where small locus changes may be significant—for clinical purposes, the smaller grid seems to provide accurate enough information to characterize the retinal sensitivity. It certainly provides more central retina-focused information on progression and prognostication as compared to a full-field static perimetry, at least in conditions affecting the central retina. In our personal experience in trials involving individuals with poor rod function, such as with forms of rod–cone dystrophy, the traditional grid makes for a very long test time, which can be frustrating for the patient, generating many unnecessary repeat tests; a faster grid can perhaps help under those circumstances—with the trade-off having less loci-specific information.

Moreover, it can be integrated into busy clinical settings, which is the main purpose of this workflow. Similarly, microperimetry is a psychophysical modality that can be easily analyzed longitudinally and one that is arguably more sensitive to small changes than visual acuity for a variety of retinal diseases, such as diabetic retinopathy,^8^ age-related macular degeneration,^9^^,^^10^ and inherited retinal diseases, where the functional deterioration may precede visible, macroscopic structural changes. Furthermore, it can be inputted into custom-made software such as the visual field modeling analyzer, providing effective topographic models of the hill of vision.^11^

The data also suggest that this is a feasible methodology and grid, and that this workflow of photopic, followed by mesopic and scotopic after 30 minutes of dark adaptation, works well. The photoreceptor bleaching caused by the flash of the color fundus image can be ultimately negated using the workflow suggested in Figure 2, since the dark adaptation comes immediately after photography—with therefore no time wasted and the entire comprehensive retinal evaluation performed in under 3 hours. Scotopic microperimetry has great potential, as shown in STGD1,^12^ revealing a faster decline in mean sensitivity in scotopic versus mesopic microperimetry. One of the strengths of the present study is that the protocol suggested herein incorporates scotopic testing in a natural workflow, after in-depth functional characterization in both photopic and mesopic testing conditions.

There is a relative lack of consensus in the literature regarding performing microperimetry with or without dilation, but the workflow we propose can be adapted easily to be used after dilation. The absence of dilation did not translate into difficulties during the test, and although we have only tested healthy individuals, it is not unfeasible to suggest that this will also be the case for individuals affected with retinal disease. An interesting study published in 2017 by Han et al.^13^ compared the threshold sensitivity and fixation stability pre- and postdilation in healthy subjects and patients affected by choroideremia. The authors found no statistically significant difference with the introduction of dilation, suggesting that it does not seem to degrade or change the performance in microperimetry.

The two macular zones explored herein are worthy of further consideration. When respect to the PMZ, the difference between the grids does not seem significant, but in the CMZ, it almost reaches statistical significance. This may be due to the reduced number of test points in the central 4 degrees, as opposed to the tight arrangement in the 68-point grid. Similarly, these points are sparser in the grid proposed herein. Another point is regarding fixation stability and BCEA, with the former highly associated with fatigue; this contributes to reliability and is even more likely to occur in the presence of disease and in longer testing times.^14^^–^^16^ Hence, it would be reasonable to hypothesize that such a shorter test time, as the one the authors are proposing, may increase the reliability of the data, particularly relating to fixation. On the other hand, given the quick succession in which the tests were performed, this could contribute to increased fatigue, which may be a limitation of this methodology. A personalized approach should be taken, with breaks between each section of the workflow optimized according to the patient.

Finally, although there is not enough evidence to suggest a single device that can universally and reliably test all patients affected with retinal disorders, the MP3-S is arguably the optimal device to date. Its fast-tracking speed translates to less adjustments required by the patient and a generally smoother testing. It may also reduce the test time, since otherwise, the test may need to be interrupted on multiple occasions by the examiner for the machine to realign the grid based on the IR imaging tracker—which is particularly relevant in the case of nystagmus and/or poor fixation. Most important, perhaps, the flexibility of being able to test individuals under three different conditions is still a unique feature.

We have not attempted to assess test–retest reliability indices. However, we only included data above the 20% reliability factor threshold, and BCEA values were very small, reflecting a very high fixation stability. Other studies have indeed determined test–retest variability and the influence of learning effects between tests with different microperimetry devices in a variety of retinal diseases^17^^–^^20^ and in healthy subjects,^7^ which were not significant. Similarly, there is a possibility that larger sample sizes would increase the statistical difference between the two grids, which may or may not have clinical relevance. More healthy subjects are needed to further extrapolate our findings in the context of a bigger cohort, as well as testing both grids in individuals affected with retinal diseases. It remains to be established how well these grids will perform in the context of retinal diseases such as cone–rod dystrophies and retinitis pigmentosa and at which stage of disease. It is not unreasonable to suggest that it could be used through all disease stages, since isolated photoreceptor (i.e., cone, rod, or mixed response) function can be assessed thoroughly, meaning, for instance, early scotopic deficits in rod–cone dystrophies, with a larger window of photopic sensitivity preservation, essentially allow for long-term follow-up. This is currently a work in progress, with further data to be published in the future.

## Supplementary Material

Supplement 1
