# Supplementary material for: In-Depth Retinal Sensitivity Assessment With the MP3 Type S Microperimeter: A Methods Study
Source: Transl Vis Sci Technol. 2024 Apr 9;13(4):14. doi: 10.1167/tvst.13.4.14 (PMC11008759; doi:10.1167/tvst.13.4.14)
Supplement: Supplement 1 [file tvst-13-4-14_s001.docx]

Jun 20, 23

Photopic/Mesopic/Scotopic testing in the MP3-S

**Working practice**

1. **Purpose**

To document and functionally characterise patients affected with inherited retinal dystrophies (IRD) by performing fundus-guided perimetry (FGP) in a photopic, mesopic and scotopic setting.

1. **Equipment**

The NIDEK MP3-S Microperimeter will be used.

1. **Procedure**

**Photopic testing**

1. The test will be performed without pupil dilation. The order will always be right eye, followed by left eye. No dark adaptation is necessary for photopic.
2. Sit the patient comfortably in the chair in the darkened room and use a patch to cover the non-tested eye. A dim light in the corner of the room may be used for the examiner to navigate as needed.
3. Make sure that the lever to the left of the MP3-S is set to ‘P’. Turn on Nidek MP3-S and open the Navis-EX software (not actually required for testing, but good to have a general overview of previous patients/exams).
4. With the touch-pen, click ‘Patients’, then ‘+New’. Enter the subject as follows:

- Patient ID: Study code followed by study ID (eg., MGT020-001);
- Leave the other options empty.

1. Select the patient. For a new baseline assessment, touch the first set of three dots ‘…’ and choose the custom configuration created named ‘MGT020 PHOTO’. If a follow-up exam instead, select ‘Exams’, click on the desired exam to be used as baseline and touch ‘Follow Up’ - the same configurations of the previous test will be applied. If the patient has never had a previous FGP test, a short practice session is recommended - in that case, touch ‘Start Practice’. If the test is with a patient who has performed it previously, choose ‘Start Exam’ instead. The parameters have all been previously set and therefore no adjustment is needed.

For reference, please check ‘MGT020 PHOTO’ test parameters section.

1. Instructions to the patient: ‘You will see a red cross in middle of the screen which should be clear. Please look at the middle of it during the entire test and let me know if you cannot see it. In just a few moments, dim dots will pop-up around the cross in the screen, which will vary in position and light intensity. If you see a dot of light, no matter how bright, dim or blurry, press your response button. Please do not search for the dots and instead fixate at the target straight ahead for the entire test. You may blink as needed, but the best time to blink is just as you are pushing the button.’
2. If the patient cannot see the fixation cross, touch ‘Configure’. The fixation target can be thickened or increased in size (in degrees). In case of the cross, it can be possibly changed to a circle. Touch ‘Done’ when finished. If still not visible, the patient can be continuously guided verbally based on the fundus image on the screen. Select the eye and adjust chin rest by touching either ‘Right’ or ‘Left’ with the touch pen. Touch OK and the automatic alignment will start. If failed, semi-automatic retina alignment will start, and the examiner has to manually define the alignment with the joystick. After alignment, focus and position check are completed, the test may start.
3. Always encourage the patient during the test and ensure the head is in the correct position.
4. At the end of the test, a fundus photo will NOT be acquired.
5. At least 5 min rest are recommended, then test the left eye (OS) in the same setting.

**Mesopic testing**

1. The test will be performed without pupil dilation. The order will always be right eye, followed by left eye. No dark adaptation is necessary for mesopic.
2. Sit the patient comfortably in the chair in the darkened room and use a patch to cover the non-tested eye. A dim light in the corner of the room may be used for the examiner to navigate as needed.
3. Make sure that the lever to the left of the MP3-S is set to ‘P’. Turn on Nidek MP3-S and open the Navis-EX software (not actually required for testing, but good to have a general overview of previous patients/exams).
4. With the touch-pen, click ‘Patients’, then ‘+New’. Enter the subject as follows:
   - - - - Patient ID: Study code followed by study ID (eg., MGT020-001);
         - Leave the other options empty.
5. Select the patient. For a new baseline assessment, touch the first set of three dots ‘…’ and choose the custom configuration created named ‘**MGT020 MESO**’. If a follow-up exam instead, select ‘Exams’, click on the desired exam to be used as baseline and touch ‘Follow Up’ - the same configurations of the previous test will be applied. If the patient has never had a previous FGP test, a short practice session is recommended - in that case, touch ‘Start Practice’. If the test is with a patient who has performed it previously, choose ‘Start Exam’ instead. The parameters have all been previously set and therefore no adjustment is needed.

For reference, please check ‘**MGT020 MESO**’ test parameters section.

1. Instructions to the patient: ‘You will see a red cross in middle of the screen which should be clear. Please look at the middle of it during the entire test and let me know if you cannot see it. In just a few moments, dim dots will pop-up around the cross in the screen, which will vary in position and light intensity. If you see a dot of light, no matter how bright, dim or blurry, press your response button. Please do not search for the dots and instead fixate at the target straight ahead for the entire test. You may blink as needed, but the best time to blink is just as you are pushing the button.’
2. If the patient cannot see the fixation cross, touch ‘Configure’. The fixation target can be thickened or increased in size (in degrees). In case of the cross, it can be possibly changed to a circle. Touch ‘Done’ when finished. If still not visible, the patient can be continuously guided verbally based on the fundus image on the screen.
3. Select the eye and adjust chin rest by touching either ‘Right’ or ‘Left’ with the touch pen. Touch OK and the automatic alignment will start. If failed, semi-automatic retina alignment will start, and the examiner has to manually define the alignment with the joystick. After alignment, focus and position check are completed, the test may start.
4. Always encourage the patient during the test and ensure the head is in the correct position.
5. At the end of the test, a fundus photo will be acquired.
6. At least 5 min rest are recommended, then test the left eye (OS) in the same setting;

**Scotopic testing**

Before to perform the Scotopic exam, remember to set the lever to the left of the MP3-S from ‘P’ to ‘S’ and to reduce the luminance of the PC monitor (by using the monitor menu).

1. The test will be performed without pupil dilation. The order will always be right eye, followed by left eye. At least 30 min of dark adaptation is required for scotopic testing.
2. Sit the patient comfortably in the chair in the complete dark room and use a patch to cover the non-tested eye.
3. Make sure that the lever to the left of the MP3-S is set to ‘S’. Turn on Nidek MP3-S and open the Navis-EX software (not actually required for testing, but good to have a general overview of previous patients/exams).
4. With the touch-pen, click ‘Patients’, then ‘+New’. Enter the subject as follows:
   - - - - Patient ID: Study code followed by study ID (eg., MGT020-001);
         - Leave the other options empty.
5. Select the patient. For a new baseline assessment, touch the first set of three dots ‘…’ and choose the custom configuration created named ‘**MGT020 SCOTO**’. If a follow-up exam instead, select ‘Exams’, click on the desired exam to be used as baseline and touch ‘Follow Up’ - the same configurations of the previous test will be applied. If the patient has never had a previous FGP test, a short practice session is recommended - in that case, touch ‘Start Practice’. If the test is with a patient who has performed it previously, choose ‘Start Exam’ instead. The parameters have all been previously set and therefore no adjustment is needed.

For reference, please check ‘**MGT020 SCOTO**’ test parameters section.

1. Instructions to the patient: ‘You will see a white ring in the middle of the screen which should be clear. Please look at the middle of it during the entire test and let me know if you cannot see it. In just a few moments, dim dots will pop-up around the circle in the screen, which will vary in position and light intensity. If you see a dot of light, no matter how bright, dim or blurry, press your response button. Please do not search for the dots and instead fixate at the target straight ahead for the entire test. You may blink as needed, but the best time to blink is just as you are pushing the button.’
2. If the patient cannot see the fixation target, touch ‘Configure’. The fixation target can be thickened or increased in size (in degrees). Touch ‘Done’ when finished. If difficulties to see the circle, the target can be changed to four crosses. If still not visible, the patient can be continuously guided verbally based on the fundus image on the screen.
3. Select the eye and adjust chin rest by touching either ‘Right’ or ‘Left’ with the touch pen. Touch OK and the automatic alignment will start. If failed, semi-automatic retina alignment will start, and the examiner has to manually define the alignment with the joystick. After alignment, focus and position check are completed, the test may start.
4. Always encourage the patient during the test and ensure the head is in the correct position.
5. At the end of the test, a fundus photo will NOT be acquired.
6. At least 2-3 min rest are recommended, then test the left eye (OS) in the same setting;

At the end of the exams acquisition (Photopic, Mesopic and Scotopic), please use the colour fundus acquired during the Mesopic testing to over impose the IR exams acquired on photopic and scotopic testing.

1. **Test Parameters**

**MGT020 PHOTO**

[General]

Exam Type: Static Microperimetry Configuration: MGT020 PHOTO

[Examination]

Pattern Type: Automatic Pattern Name: MGT020 Photopic

Strategy: 4-2 (fast) Duration: 200 msec

Refinement: Yes Recheck: Yes Pre-Test: Yes

Perimetric scale: 31.4 asb – White on White – 34 dB (0.003 asb – 24 dB for scotopic)

Stimulus: Goldmann III (Goldmann V for scotopic) Attenuation: 27 dB

[Fixation target]

Shape: Single cross Color: Red Thickness: 0.20° Brightness: Max Size: 1.00°

circle for scotopic (white, max, 3.0)

[Automatism]

Pupil Alignment: Automatic Retina Alignment: Automatic

Retina Focusing: Automatic IR Control: Automatic

Optional Color Fundus Image: No Auto shot: No

**MGT020 MESO**

[General]

Exam Type: Static Microperimetry Configuration: MGT020 MESO

[Examination]

Pattern Type: Automatic Pattern Name: MGT020 Mesopic

Strategy: 4-2 (fast) Duration: 200 msec

Refinement: Yes Recheck: Yes Pre-Test: Yes

Perimetric scale: 4 asb – White on White – 34 dB (by flagging the ‘MP-1 scales’ box)

Stimulus: Goldmann III Attenuation: 27 dB

[Fixation target]

Shape: Single cross Color: Red Thickness: 0.20° Brightness: Max Size: 1.00°

[Automatism]

Pupil Alignment: Automatic Retina Alignment: Automatic

Retina Focusing: Automatic IR Control: Automatic

Optional Color Fundus Image: Yes Auto shot: Yes

Reduce the flash intensity to ‘4’, otherwise, the flash is too bright. If the fundus photo is dark and needs more lighting, another separate photo can be taken after the test with a higher flash intensity

**MGT020 SCOTO**

[General]

Exam Type: Scotopic Static Microperimetry Configuration: MGT020 SCOTO

[Examination]

Pattern Type: Automatic Pattern Name: MGT020 Scotopic

Strategy: 4-2 (fast) Duration: 200 msec

Refinement: Yes Recheck: Yes Pre-Test: Yes

Perimetric scale: 0.003 asb – White on White – 24 dB

Stimulus: Goldmann V Attenuation: 19 dB

[Fixation target]

Shape: Circle Color: White Thickness: 0.20° Brightness: Max Radius: 3.00°

[Automatism]

Pupil Alignment: Automatic Retina Alignment: Automatic

Retina Focusing: Automatic IR Control: Automatic

Optional Color Fundus Image: No Auto shot: No
